# Bornyl-Containing Derivatives of Benzyloxyphenylpropanoic Acid as FFAR1 Agonists: In Vitro and In Vivo Studies

**DOI:** 10.3390/pharmaceutics15061670

**Published:** 2023-06-07

**Authors:** Darya A. Pon’kina, Sergey O. Kuranov, Mariya K. Marenina, Yulia V. Meshkova, Nataliya A. Zhukova, Mikhail V. Khvostov, Olga A. Luzina, Tatiana G. Tolstikova, Nariman F. Salakhutdinov

**Affiliations:** N. N. Vorozhtsov Novosibirsk Institute of Organic Chemistry, Siberian Branch of the Russian Academy of Sciences, 9, Akademika Lavrentieva Ave., Novosibirsk 630090, Russia; s.o.kuranov@chemomsu.ru (S.O.K.); fominamk@gmail.com (M.K.M.); meshkova_29@mail.ru (Y.V.M.); gna2004@ngs.ru (N.A.Z.); khvostov@nioch.nsc.ru (M.V.K.); luzina@nioch.nsc.ru (O.A.L.); tolstiktg@nioch.nsc.ru (T.G.T.); anvar@nioch.nsc.ru (N.F.S.)

**Keywords:** bornyl derivatives, FFAR1 agonist, hypoglycemic activity, hepatoprotective effect, OGTT, diabetes mellitus

## Abstract

Type 2 diabetes mellitus (T2DM) is one of the most common chronic diseases worldwide. Several classes of hypoglycemic drugs are used to treat it, but various side effects limit their clinical use. Consequently, the search for new anti-diabetic agents remains an urgent task for modern pharmacology. In this investigation, we examined the hypoglycemic effects of bornyl-containing benzyloxyphenylpropanoic acid derivatives (QS-528 and QS-619) in a diet-induced model of T2DM. Animals were given the tested compounds per os at a dose of 30 mg/kg for 4 weeks. At the end of the experiment, compound QS-619 demonstrated a hypoglycemic effect, while QS-528 showed hepatoprotection. In addition, we performed a number of in vitro and in vivo experiments to study the presumed mechanism of action of the tested agents. Compound QS-619 was determined to activate the free fatty acid receptor-1 (FFAR1) similarly to the reference agonist GW9508 and its structural analogue QS-528. Both agents also increased insulin and glucose-dependent insulinotropic polypeptide concentrations in CD-1 mice. Our results indicate that QS-619 and QS-528 are probably full FFAR1 agonists.

## 1. Introduction

**Figure 1:**
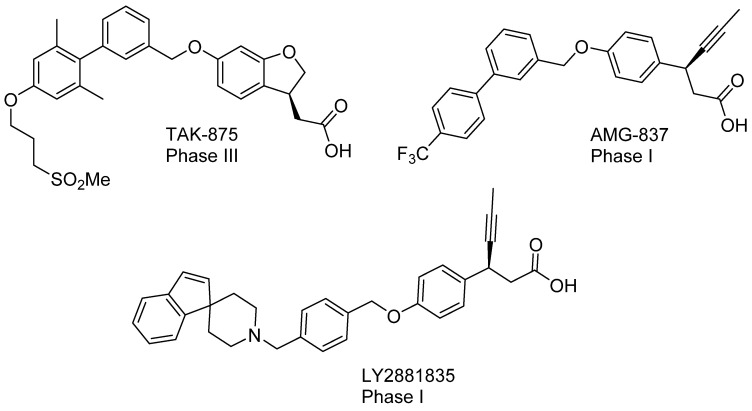
FFAR1 agonists.

Type 2 diabetes mellitus (T2DM) is a chronic multisystem disease characterized by reduced peripheral tissue sensitivity to insulin (insulin resistance) and/or relative pancreatic β-cell dysfunction [1,2]. Hyperglycemia occurs due to an inability of target tissues to adequately utilize glucose and insulin’s reduced ability to inhibit gluconeogenesis, lipolysis and glycogenesis [3,4]. One of the areas of great interest currently is the role of free fatty acids in gluconeogenesis. The free fatty acid receptor 1 (FFAR1, formerly GPR40) is a potential target for the treatment of type 2 diabetes mellitus (T2DM). This receptor is widely expressed in pancreatic β-cells and intestinal enteroendocrine cells [5]. Endogenous free fatty acids and therapeutic FFAR 1 agonists, by activating the corresponding receptor on pancreatic β-cells, stimulate insulin secretion in a glucose-dependent way. Because of this, FFAR 1 agonists, such as GLP-1 agonists and DPP4 inhibitors, have no risk of hypoglycemia [6]. Numerous FFAR1 agonists have been developed. Of these, only a few compounds have reached the clinical trials. The most studied of these are the following synthetic agonists: TAK-875 (has reached phase 3 of clinical trials (CT)), LY2881835 (has reached phase 1 CT), AMG 837 (has reached phase 1 CT) (Figure 1) [7,8]. However, their further study was discontinued due to hepatotoxicity. It Is worth noting that all these compounds were FFAR1 partial agonists and stimulated insulin secretion directly, only through interaction with the FFA1 receptor on pancreatic β-cells [9]. FFAR1 full agonists are additionally able to stimulate insulin secretion indirectly by increasing the secretion of the incretins—glucagon-like peptide (GLP-1) and glucose dependent insulinotropic polypeptide (GIP) by activation of FFAR1 in enteroendocrine K- and L-cells, respectively [10]. This makes them more effective at controlling glycaemia than partial agonists, as well as reducing food intake and body weight [7,10]. Furthermore, in addition to their antidiabetic action, FFAR1 full agonists have been found to have therapeutic effects against non-alcoholic fatty liver disease (NAFLD) [11]. NAFLD is currently the most frequent liver disease in the world, and fatty acids play a large role in this pathologic condition [12,13].

**Figure 2:**
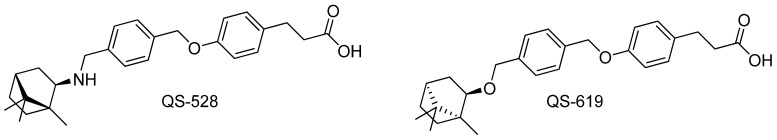
Bornyl-containing derivatives of benzyloxyphenylpropanoic acid.

We have previously demonstrated that an isobornilamine derivative (QS-528) (Figure 2) is an FFA1 receptor agonist and exhibits hypoglycemic and hepatoprotective effects in in vivo tests in mice in different doses [14]. Its structural analogue, the borneol derivative (QS-619) (Figure 2), previously demonstrated hypoglycemic effects in a genetic model of T2DM at a dose of 30 mg/kg [15]. In the present work, we investigated the hypoglycemic effects of both compounds in a diet-induced model of T2DM and studied in more depth the mechanisms of their action.

## 2. Materials and Methods

### 2.1. Investigated Compound

Compounds QS-528 and QS-619 were synthesized according to previously described methods [14,15] and used in the experiment as a hydrochloride salt and in free form, respectively.

### 2.2. Animals

Male CD-1 and C57Bl/6J mice weighing 25–30 g were used in the experiment. Animals were obtained from the vivarium of the Institute of Cytology and Genetics of the Siberian Branch of the Russian Academy of Sciences and kept under standard vivarium conditions with free access to water and standard granulated chow in a humidity- and temperature-controlled room on a 12/12 h light–dark cycle. All manipulations with animals were carried out in strict accordance with the legislation of the Russian Federation, a decree of the Ministry of Health of the Russian Federation No. 199 n of 1 April 2016 and the provisions of Directive 2010/63/EU of the European Parliament and of the Council of the European Union of 22 September 2010 on the protection of animals used for scientific purposes. The experiment was approved by the Ethics Committee of the N. N. Vorozhtsov Institute of Organic Chemistry SB RAS (*p*-01-04.2022-14).

### 2.3. Oral Glucose Tolerance Test (OGTT)

Test compounds according to 2.4 were given 30 min prior to an oral glucose load (2.5 g/kg). Blood glucose concentration was measured with a ONE TOUCH Select glucose meter (LIFESCAN Inc., Milpitas, CA, USA) before glucose introduction (0 min) and 30, 60, 90, 120 min after glucose introduction. The area under the glycemic curve was calculated using Tai’s mathematical model [16].

### 2.4. Diet-Induced Model of Type 2 Diabetes Mellitus

Male C57Bl/6J mice were kept on a high-fat (36%) and high-carbohydrate (37%) diet (HFD) for 7 months. Once animals reached impaired glucose tolerance (according to OGTT), they were divided into 5 groups (*n* = 6–8 mice). The animals were then given the test compounds daily per os for 4 weeks and the diet was not changed. Group 1 “Intact control” mice received H_2_O distilled + 0.5% Tween 80; Group 2 “Negative control” received HFD + H_2_O distilled + 0.5% Tween 80; Group 3 “Positive control” received HFD + metformin (CAS 1115-70-4 Acros Organics, Geel, Belgium) 250 mg/kg + H_2_O distilled; Group 4 received HFD + QS-528 30 mg/kg + H_2_O distilled + 0.5% Tween 80; Group 5 received HFD + QS-619 30 mg/kg + H_2_O distilled + 0.5% Tween 80. OGTT was performed on days 14 and 28 of the experiment. At the end of the experiment (day 30), the mice were decapitated, blood was taken for biochemical analysis and the liver was harvested for histological examination.

### 2.5. Biochemical Assays

Blood was centrifuged at 1640 g for 15 min to obtain serum. Triglycerides (TG) and alanine aminotransferase (ALT) were determined using standard diagnostic kits (Vector-Best, Novosibirsk, Russia) and a Multiscan Ascent photometer (Thermo Labsystems, Helsinki, Finland).

### 2.6. Histological Liver Examination

The liver was fixed in 10% neutral buffered formalin for 7 days, and then standard dehydration in ascending ethanol concentrations and xylene was carried out. All samples were embedded in paraffin on an AP 280 workstation using Histoplast (Thermo Fisher Scientific, Waltham, MA, USA) with a melting point of 58 °C. Tissue slices with a thickness of 4.5 μm were prepared on a rotational NM 335E microtome with disposable interchangeable blades. The slices were stained with periodic acid–Schiff, hematoxylin, eosin and orange G and then were examined under a light microscope at a magnification of ×200.

### 2.7. In Vitro FFAR1 Activation Assay

A standard FFAR1 (GPR40) Reporter Assay Kit (Cat. No. 601190, Cayman Chemical Ann Arbor, MI, USA) was used in the study. All compounds were tested at a concentration of 10 µM. Sample preparation and all procedures were carried out in accordance with the manufacturer’s instructions. A known FFAR1 agonist, GW9508 (included in the kit), was used as a positive control. A ClarioStar multimodal reader (BMG Labtech, Ortenberg, Germany) was used for the assay. The ability of compounds QS-528 and QS-619 to activate the FFA1 receptor is presented as a percentage and evaluated relative to the maximum potency of GW9508.

### 2.8. In Vitro Inhibition of DPP4 Assay

A fluorometric screening assay kit (DPP (IV) Inhibitor Screening Assay Kit, Cayman Chemical, Ann Arbor, MI, USA) was used to determine the inhibitory activity of QS-528 and QS-619 against DPP4. Sitagliptin (as part of the kit) was used as a positive control. The compounds were diluted in dimethylsulphoxide (DMSO (Reagent Component, Moscow, Russia)) and tested at a final concentration of 100 µM (in 10% DMSO). The assay was based on the liberation of AMC (7-amino-4-methyl-coumarin) from the DPP4 substrate, Gly-Pro-AMC. Fluorescence changes resulting from the cleavage of the DPP4 molecule were measured at excitation and emission wavelengths of 350 and 450 nm using ClarioStar (BMG Labtech, Ortenberg, Germany). The percentage inhibition of DPP4 activity was calculated relative to baseline activity (control group, without inhibitors) using the formula that follows: %inhibition = (baseline activity − activity with inhibitor)/baseline activity × 100.

### 2.9. Insulin ELISA Examination

We used male CD-1 mice, which were divided into 4 groups (*n* = 5). Animals were given QS-528 and QS-619 compounds per os at a dose of 30 mg/kg after 12 h fasting. Vildagliptin (Galvus, Novartis, Moscow, Russia) per os at a dose of 10 mg/kg was used as a positive control. All the compounds tested were premixed with 2 drops of Tween 80 and dissolved in distilled H_2_O. Animals in the control group were given only water with Tween. We performed an oral glucose load (2.5 g/kg) 30 min after administration of the test compounds. Blood in the amount of 0.1 mL was collected from the animal’s tail before administration of the compounds and then 15, 30, 60 min after glucose loading. Blood samples were centrifuged for 15 min at 1640 g after coagulation (at least 30 min) to separate the serum. Serum samples were frozen at −80 °C for further assay. The standard ELISA kit (Cat. No. EZRMI-13K, Millipore, Merck KGaA, Darmstadt, Germany) was used to determine insulin concentrations. Sample preparation and all procedures were performed according to the manufacturer’s instructions. A Multiscan Ascent photometer (Thermo Labsystems, Helsinki, Finland) was used for the analysis.

### 2.10. GIP ELISA Examination

Male CD-1 mice, divided into 4 groups (*n* = 7), were used for the experiment. Compounds QS-528 and QS-619 were administered per os at a dose of 30 mg/kg. Vildagliptin (Galvus, Novartis, Moscow, Russia) was used as a positive control per os at a dose of 10 mg/kg. All test compounds were premixed with two drops of Tween 80, dissolved in distilled H_2_O and administered to fasting (12 h) animals. Mice in the control group were given only distilled H_2_O with 2 drops of Tween 80. We performed an oral glucose load (2.5 g/kg) 30 min after administration of the test compounds. Next, 0.1 mL of blood was collected from the animal’s tail before the administration of the compounds and then 5 and 10 min after glucose loading. Blood samples were centrifuged for 15 min at 1640 g after coagulation (at least 30 min) to separate serum. Serum samples were frozen at −80 °C for further assay. A standard ELISA kit (Cat. No. 81527, Crystal Chem, USA) was used to determine serum GIP concentrations. Sample preparation and all procedures were performed according to the manufacturer’s instructions. A Multiscan Ascent photometer (Thermo Labsystems, Helsinki, Finland) was used for the analysis.

### 2.11. Toxicology Study

In order to determine the acute toxicity (LD_50_), QS-619 was administered per os to CD-1 mice (*n* = 8) at a single dose of 1000 mg/kg. Animals’ behavior, body weight and lethality were evaluated over the next 10 days.

### 2.12. Statistical Analysis

Statistical analysis was performed in Statistica 10.0 software using one-way ANOVA, followed by the Fisher LSD test for multiple comparisons. All data are presented as mean ± SEM (standard error of the mean). Differences with *p* ≤ 0.05 were considered statistically significant.

## 3. Results

### 3.1. OGTT in C57Bl6/J Mice

**Figure 3:**
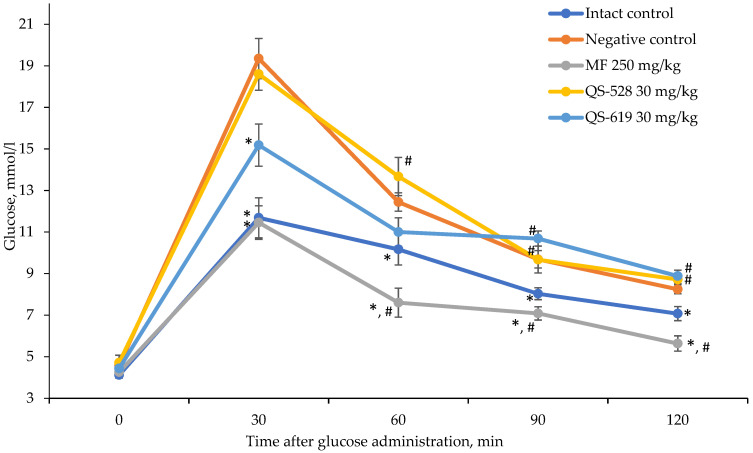
OGTT in C57Bl/6J mice after 2 weeks of the experiment. * *p* ≤ 0.05 as compared to the negative control (C57Bl/6J); ^#^ *p* ≤ 0.05 as compared to the intact control. MF: metformin.

According to the oral glucose tolerance test (OGTT) performed 2 weeks after the experiment start, impaired glucose tolerance was retained in the negative control group. This confirms blood glucose levels at all time points compared to the intact control group (Figure 3). The glycemic profile of compound QS-528 was almost identical to the negative control group, whereas compound QS-619 significantly reduced the blood glucose concentration in experimental animals 30 min after glucose load. In this experiment, only the metformin group showed a statistically significant reduction in glucose at all time points (Figure 3).

**Figure 4:**
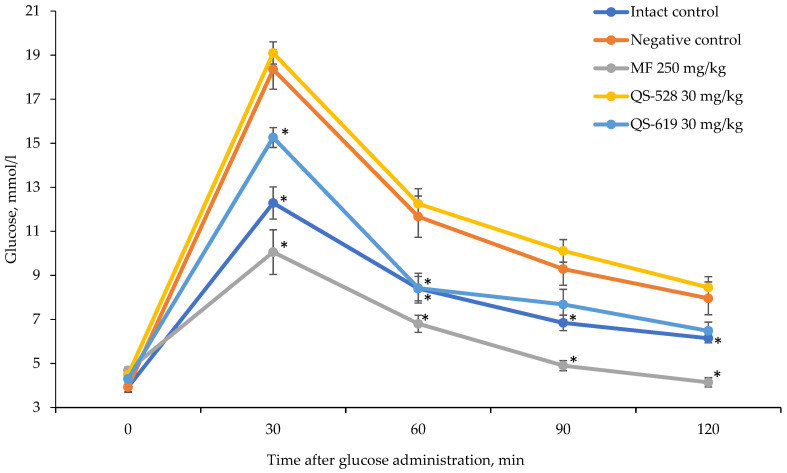
OGTT in C57Bl/6J mice after 4 weeks of the experiment. * *p* ≤ 0.05 as compared to the negative control (C57Bl/6J). MF: metformin.

**Figure 5:**
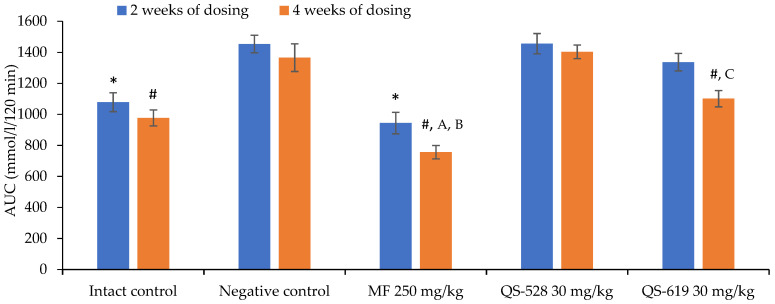
OGTT in C57Bl/6J mice after 4 weeks of the experiment. * *p* ≤ 0.05 as compared to the negative control after 2 weeks of experiment; ^#^ *p* ≤ 0.05 as compared to the negative control after 4 weeks of experiment; ^A^ *p* ≤ 0.05 as compared to the intact control after 4 weeks of experiment; ^B^ *p* ≤ 0.05 as compared to metformin after 2 weeks of experiment; ^C^ *p* ≤ 0.05 as compared to QS-619 after 2 weeks of experiment. MF: metformin.

The experiment was continued for 2 additional weeks, at the end of which OGTT was carried out again. Impaired glucose tolerance remained unchanged in mice of the negative control group (Figure 4). A hypoglycemic effect was found in mice treated with QS-619. This can be seen both in the glycemic curve profile when compared to that of intact control (Figure 4) and according to the area under the glycemic curve (AUC) (Figure 5). Compound QS-528 showed no activity (Figure 4 and Figure 5). The administration of metformin for 4 weeks improved the mice’s glucose tolerance more significantly (Figure 5).

### 3.2. A Biochemical Blood Assay

**Figure 6:**
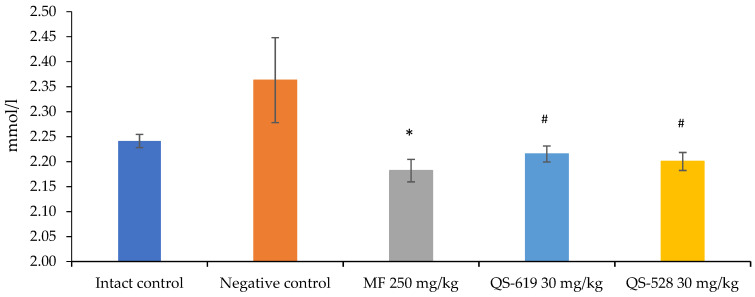
Triglyceride (TG) concentrations in the mice’s blood after 4 weeks of experiment. * *p* ≤ 0.05 as compared to the negative control; ^#^ *p* = 0.05–0.1 as compared to the negative control. MF: metformin.

The triglyceride (TG) and alanine aminotransferase (ALT) concentrations in the blood were assessed. A significant decrease in TG concentrations was found in the group treated with metformin (Figure 6). Compounds QS-528 and QS-619 showed a downward trend in this biochemical parameter (Figure 6).

**Figure 7:**
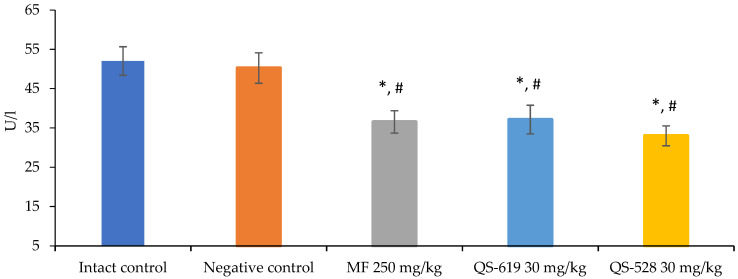
Alanine aminotransferase (ALT) concentrations in the blood from the mice after 4 weeks of experiment. * *p* ≤ 0.05 as compared to the intact control; ^#^ *p* ≤ 0.05 as compared to the negative control.

Metformin and the compounds QS-528 and QS-619 demonstrated the reduction of the blood ALT concentration of mice compared to both intact and negative controls (Figure 7).

### 3.3. Histological Examination

**Figure 8:**
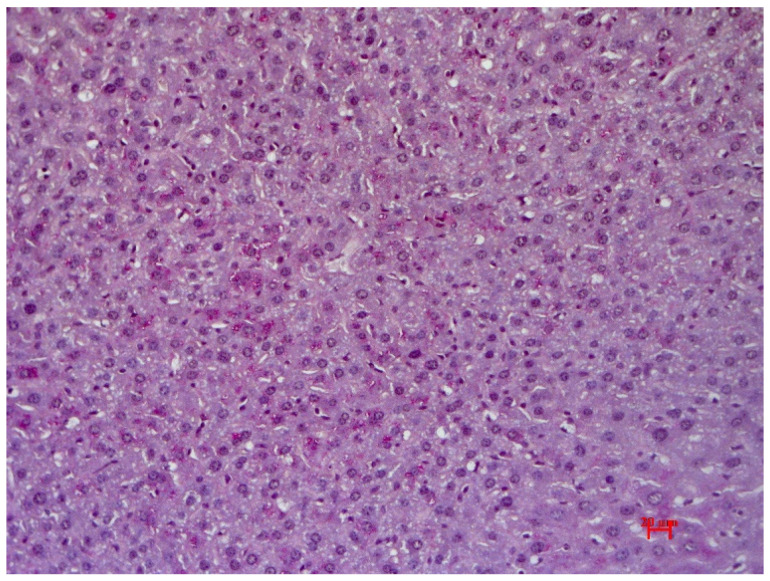
The liver of control mice without pathological changes. Glycogen in single hepatocytes. Staining with PAS hematoxylin orange G, ×200.

Animals in the control group showed preserved liver architectonics, bile capillary structure, veins and arteries; all were unchanged in mice of the control group. Moreover, no signs of pathological infiltration, dystrophy or fibrosis were found. Uneven glycogen distribution in the form of dust-like granularity was observed during Periodic acid–Schiff staining (PAS staining) (Figure 8).

**Figure 9:**
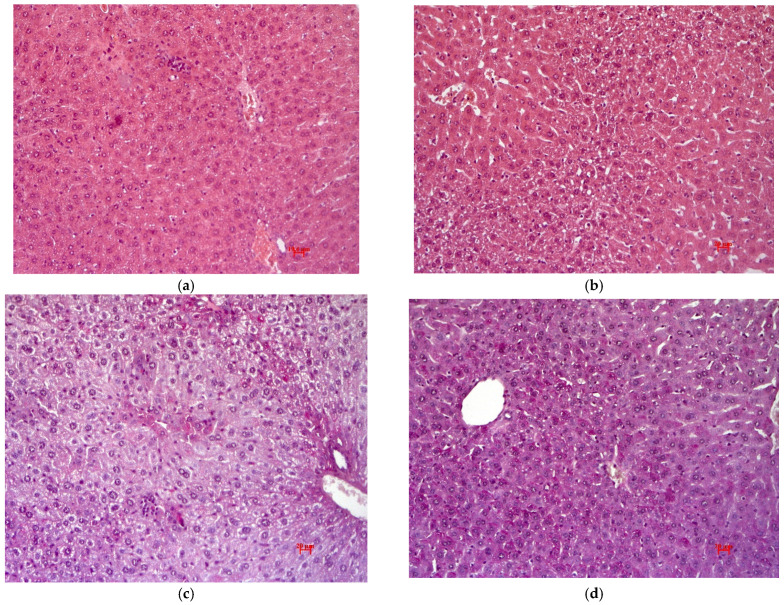
Histological examination of mouse livers after 4 weeks of the experiment. (**a**) Negative control. Fatty dystrophy and small focal necrosis of hepatocytes infiltrated by mononuclear cells. Staining with hematoxylin-eosin, ×200. (**b**) Positive control (metformin). Staining with hematoxylin-eosin, ×200. (**c**) QS-619. Fatty dystrophy of hepatocytes, perisinusoidal fibrosis. Staining with PAS-hematoxylin orange G, ×200. (**d**) QS-528. Uneven glycogen distribution in hepatocytes. Staining with PAS-hematoxylin orange G, ×200.

The development of fatty hepatosis was observed in animals with diet-induced T2DM. Multivesicular lipid infiltration; focal necrosis of hepatocytes infiltrated by macrophages and mononuclear leukocytes; and hepatic bar dyscomplexation in periportal zones were found (Figure 9a). No glycogen was detected. Metformin treatment did not recover these abnormalities (Figure 9b).

Degenerative-necrotic and hemodynamic abnormalities in the liver were retained in mice treated with QS-619. A pronounced small vesicular lipid infiltration of hepatocytes, as well as focal necrosis, infiltrated by monocytes and macrophages were detected (Figure 9c). No glycogen was detected.

Liver abnormalities were less pronounced in mice treated with compound QS-528. There was a regression of fatty hepatosis. No marked infiltrative-necrotic and hemodynamic changes were detected. An irregular distribution of glycogen in hepatocytes was observed (Figure 9d).

### 3.4. In Vitro FFAR1 Activation and DPP IV Inhibition Assay

**Figure 10:**
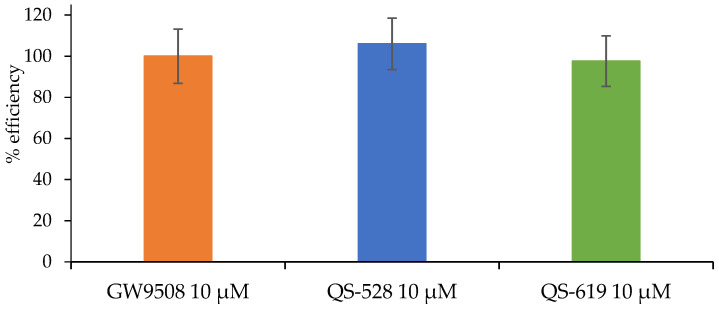
The ability of compounds QS-528 and QS-619 to activate FFAR1.

QS-528 demonstrated FFAR1 activation at 10 µM in our earlier work [14]. Here we evaluated this effect for the QS-619 which is a structural analogue of QS-528 at the same concentration (Figure 10). The activities of the known agonist GW9508 and the compound QS-528 [16] were also measured and used for comparison. Based on our results, it can be concluded that QS-619 also exhibits high affinity for the FFA1 receptor.

In addition, we studied the ability of QS-528 and QS-619 to inhibit the DPP4 enzyme in vitro at a concentration of 100 µM. However, these compounds have not been shown to reliably inhibit the enzyme.

### 3.5. Measurement of Serum Insulin and GIP Concentrations

**Figure 11:**
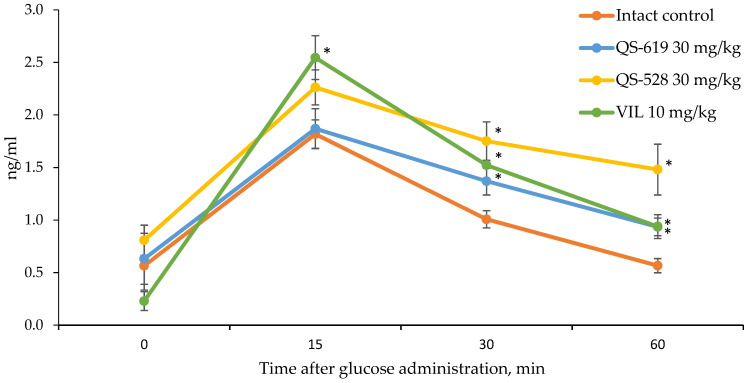
Blood insulin concentration in CD-1 mice. * *p* ≤ 0.05 as compared to the intact control. VIL: vildagliptin.

Activation of the FFA1 receptor is known to result in a glucose-dependent increase in insulin secretion [17]. We therefore carried out an experiment to determine the concentration of this hormone in the blood of CD-1 mice after single administration of QS-528 and QS-619. Both compounds at a dose of 30 mg/kg were found to stimulate insulin secretion as well as the reference compound vildagliptin at a dose of 10 mg/kg (Figure 11).

**Figure 12:**
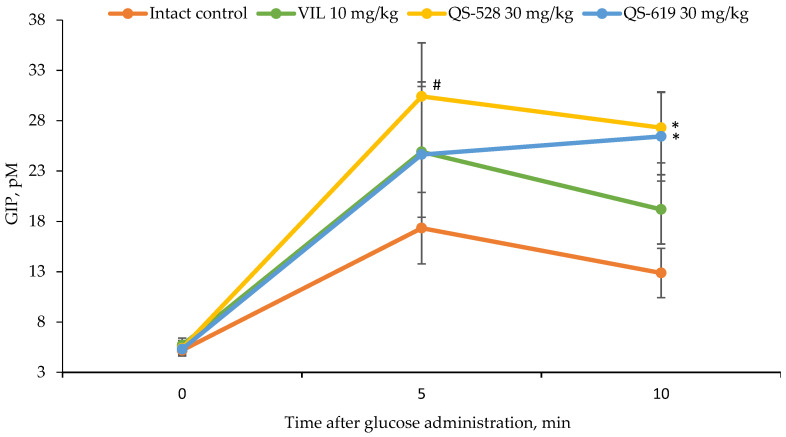
GIP concentrations in the blood of CD-1 mice. * *p* ≤ 0.05 as compared to the intact control, # *p* = 0.1–0.05 as compared to the intact control. VIL: vildagliptin.

In addition to stimulating insulin secretion, activation of the FFAR1 may promote GLP-1 and GIP secretion via intestinal enteroendocrine cells [18]. According to this, we investigated the ability of our compounds to increase the concentration of GIP in the mice’s blood. Compounds QS-528 and QS-619 were found to increase the plasma GIP concentration 5 and 10 min after glucose administration, confirming their presumed mechanism of action (Figure 12).

### 3.6. Acute Toxicity Study

**Figure 13:**
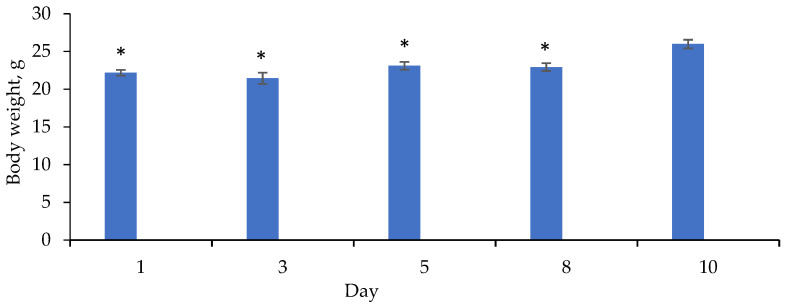
Body weight of CD-1 mice (*n* = 10) after a single administration of QS-619 at a dose of 1000 mg/kg. * *p* ≤ 0.05 as compared to the mouse weight after 10 days of QS-619 introduction.

Compound QS-619 at a dose of 1000 mg/kg was found to have no negative effect on the change in body weight of mice over the 10 days of the experiment (Figure 13). We did not observe any animal deaths or changes in appearance or behavior.

## 4. Discussion

In this work, we investigated the hypoglycemic effects of two bornyl-containing benzyloxyphenylpropanoic acid derivatives (Figure 2) at a dose of 30 mg/kg in a model of diet-induced T2DM. Only QS-619 was found to have hypoglycemic activity (Figure 4 and Figure 5). The most pronounced effect was seen at the end of the 4th week of the experiment. The effect of QS-619 appears to be related to an improved peripheral tissue sensitivity to insulin. Compound QS-528 showed no activity in this experiment (Figure 5). However, an earlier study in C57Bl/6^Ay^ mice noted the hyperglycemic effect of this compound at a single introduction, which subsequently decreased to approximately intact control levels in an OGTT conducted 2 weeks after the start of the study [15]. The absence of any hyperglycemic action of QS-528 in C57Bl/6J mice in the present experiment is probably due to less severe fatty liver dystrophy and impaired lipid metabolism, which is clearly evident in C57Bl/6^Ay^ mice [19,20]. Earlier in the same mice, the compound QS-528 was found to exhibit hepatoprotective effects [15], which were also confirmed in our experiment with biochemical (reduction of ALT) (Figure 7) and histological examination of the liver (regression of fatty hepatosis) (Figure 9d). In addition to a decrease in ALT, a tendency to lower blood TG levels in mice was found when QS-528 and QS-619 were administered (Figure 6). Consequently, it can be assumed that the compounds tested improve the lipid profile and that with longer administration this would decrease even further.

A biochemical investigation also revealed a decrease in TG concentrations in mice treated with Metformin (Figure 6). This is due to a selective brown adipose tissue-mediated increase in VLDL-TG uptake/lipolysis and subsequent mitochondrial fatty acid oxidation [21]. In addition, Metformin, along with QS-528 and QS-619, reduced ALT levels (Figure 7). This means that these substances probably reduce nuclear DNA damage and subsequent cell death in the liver of C57Bl/6J mice when kept on a high-calorie diet [22,23]. However, unlike QS-528, Metformin had no effect on improving liver condition in mice (Figure 9b).

After determining the hypoglycemic action, we studied the mechanism of action of the compounds QS-528 and QS-619. An earlier study by Kuranov et al. found QS-528 to be an FFAR1 agonist [14]. As QS-619 is its structural analogue and can therefore potentially bind to this receptor, we studied its ability to activate FFAR1. Compound QS-619, like QS-528, was found to be an agonist (10 μM) for this receptor in an in vitro study (Figure 10). As FFAR1 agonists are known to stimulate insulin and GIP secretion [10], we had to evaluate this effect for the compounds tested. Experiments conducted have confirmed this assumption (Figure 11 and Figure 12). Inhibition of DPP4 can also affect the increase of GIP concentration in blood [24]; however, we showed that studied compounds do not possess it. Thus, increased insulin and GIP concentrations probably indicate that QS-528 and QS-619 are full FFAR1 agonists [10]. It is worth noting that the dynamics of changes in the concentration of these hormones after the administration of the test agents and vildagliptin are different, which is probably due to their different mechanisms of action [25,26].

Only two FFAR1 agonists with hepatoprotective effects have been found in the literature: SCO-267 [11] and docosahexaenoic acid [27]. Both compounds significantly reduced liver steatosis in mice caused by a high-fat diet, and SCO-267 also reduced collagen, TG and ALT production [28]. Like the compound SCO-267, QS-528 also resolved fatty liver dystrophy in mice in our experiment (Figure 9d) and reduced TG (Figure 6) and ALT concentrations (Figure 7). The observed hepatoprotective effect was also found earlier in a tetrachlomethane-induced model of liver injury [29]. It is worth noting that the compound SCO-267 was initially found to have hypoglycemic effects [30], followed by hepatoprotective effects [11]. We, in turn, investigated QS-528 in a similar way, but no hypoglycemic effect was detected in this compound in a model of diet-induced T2DM (Figure 5). The mechanism of the hepatoprotective effect of QS-528 is not known, but it can be assumed that, in a model of metabolic liver damage, it resolves hepatic steatosis through stimulation of the AMP-activated protein kinase (AMPK) signaling pathway as other FFAR1 agonists [31].

We found no adverse effects of QS-619 at a dose of 1000 mg/kg in mice in an acute toxicity test (Figure 13). This suggests that its LD_50_ is significantly higher than this dose. Consequently, compound QS-619 is safe for long-term use, an extremely important characteristic of a potential drug [32]. The acute toxicity of compound QS-528 was studied previously at a dose of 1000 mg/kg and similar results were obtained [29].

From the results obtained, it can be concluded that the bornyl-containing benzyloxyphenylpropanoic acid derivatives studied in this work are most likely full FFAR1 agonists, as they stimulate insulin and GIP secretion. The compound QS-619 has a hypoglycemic effect, while QS-528 has a hepatoprotective effect.

## 5. Conclusions

In this work, we examined bornyl-containing benzyloxyphenylpropanoic acid derivatives for their hypoglycemic effect at a dose of 30 mg/kg in a diet-induced model of T2DM for four weeks. At the end of the experiment, compound QS-619 demonstrated a prominent hypoglycemic effect, whereas QS-528 demonstrated hepatoprotective action. Both compounds reduced ALT concentrations and tended to reduce TG levels in the mice’s blood. Both compounds were found to activate the FFA1 receptor and increase insulin and GIP concentrations. In an acute toxicity study, QS-619 at a single dose of 1000 mg/kg demonstrated no animal death, making it safe for long-term use along with the compound QS-528.

## Data Availability

Not applicable.
